# Designing Smartly: Understanding the Crystallinity of Melt Electrowritten Scaffolds

**DOI:** 10.1002/elsc.70020

**Published:** 2025-04-14

**Authors:** Piotr Stanisław Zieliński, Zhaohang Zhang, Ilaria Squillante, Guillermo Monreal Santiago, Marcus Koch, Giuseppe Portale, Marleen Kamperman, Anastasiia Krushynska, Małgorzata Katarzyna Włodarczyk‐Biegun

**Affiliations:** ^1^ Polymer Science – Zernike Institute for Advanced Materials University of Groningen Groningen the Netherlands; ^2^ Computational Mechanical and Materials Engineering – Engineering and Technology Institute Groningen University of Groningen Groningen the Netherlands; ^3^ Physical Chemistry of Polymeric and Nanostructured Materials – Zernike Institute for Advanced Materials University of Groningen Groningen the Netherlands; ^4^ Dutch Polymer Institute Eindhoven the Netherlands; ^5^ INM‐ Leibniz Institute for New Materials Saarbrücken Germany; ^6^ Biofabrication and Bio‐Instructive Materials Biotechnology Center The Silesian University of Technology Gliwice Poland

**Keywords:** 3D printing, additive manufacturing, crystallinity, finite element analysis, tissue engineering

## Abstract

Melt Electrowriting (MEW) is a powerful technique in tissue engineering, enabling the precise fabrication of scaffolds with complex geometries. One of the most important parameters of MEW is collector speed, which has been extensively studied in relation to critical translation speed. However, its influence on crystallinity was overlooked. Crystallinity is crucial for the mechanical properties and degradation behavior of the scaffolds. Therefore, in this study, we present how printing affects the crystallinity of fibers and the resulting mechanical properties of MEW scaffolds. In systematic analysis, we observed a significant reduction in scaffold crystallinity with increased speed, as evidenced by wide‐angle X‐ray scattering. This decrease in crystallinity was attributed to differences in cooling rates, impacting the polycaprolactone molecular orientation within the fibers. By using tensile testing, we observed the decrease in scaffold Young's modulus with increasing collector speed. Given the relation between crystallinity and mechanical properties of the material, we developed a finite element analysis model that accounts for changes in crystallinity by employing distinct bulk Young's modulus values to help characterize scaffold mechanical behavior under tensile loading. The model reveals insights into scaffold stiffness variation with different architectural designs. These insights offer valuable guidance for optimizing 3D printing to obtain scaffolds with desired mechanical properties.

## 1 Introduction

Serious diseases and injuries in the body often require tissue regeneration or replacement. With the constant scientific and technological progress, tissue engineering and regenerative medicine fields are rapidly growing. One of the most common approaches for the successful reconstruction of damaged tissues is the use of scaffolds. The scaffolds can be fabricated using additive manufacturing methods, which offer many benefits such as customization, precision, and cost‐effectiveness [1, 2]. The most commonly used approaches for tissue engineering are extrusion‐based and include electrospinning, fused deposition modeling (FDM), bioprinting, and, more recently, Melt Electrowriting (MEW) [3, 4, 5].

MEW is a 3D printing method that combines the principles, and benefits, of FDM and electrospinning, allowing for the production of high‐resolution fibrillar scaffolds [6]. With MEW, polymer fibers at the micro level can be printed due to the use of an electric field between the nozzle and the collector plate, allowing to draw a melted polymer jet. Thanks to the controlled movement of the nozzle or the collector plate, scaffolds with precisely deposited fibers can be printed into different geometries [4, 7]. With its high resolution and flexibility of possible designs, MEW constantly gains popularity, allowing to reconstruct complex architectures of native tissues. For example, MEW was applied to recreate hierarchical (multilayer) tissues, such as human trabecular meshwork [8] and skin [9] with heterogeneous geometries, and the resulting mechanical properties of different layers within the scaffolds. In another study, Castilho et al. used MEW meshes for cardiac regeneration and demonstrated increased cardiac‐related marker expressions, when cells were cultured on hexagonal‐shaped pore scaffolds, compared to rectangular pore shape [10], indicating the importance of scaffold architecture.

The overall architecture of MEW scaffolds can be fine‐tuned to meet the specific mechanical and biological requirements for tissue engineering applications. This can be done by printing different patterns and adjusting printing parameters. For instance, variations in collector speed, applied pressure, or nozzle diameter significantly influence fibers thickness, pore shape, and accuracy of deposited fibers [11, 12]. The influence of various printing parameters on MEW scaffold properties has been explored extensively [13], particularly emphasizing the significance of collector speed. Adjusting the collector speed is a common strategy to alter the fiber diameter of printed scaffolds [14, 15]. Another important aspect of the collector speed is the critical translation speed (CTS), defined by Dalton et al. as a speed when the collector and jet speeds match resulting in printing straight lines [16]. Printing with collector speeds above CTS causes an increased lag of the polymer jet and smaller precision when changing the direction of the printing path [17]. Below the CTS, the jet maintains a well‐defined shape, however, as it lands on the collector plate, the liquid coiling effect results in a sinusoidal pattern instead of the straight line [18]. Additionally, research suggests that collector speed plays a crucial role in determining extruded fiber properties, including fiber topography and microstructure [19].

The microstructure, influenced by crystallinity, may significantly affect the bulk properties of printed scaffolds, and consequently, mechanical performance and biocompatibility, as demonstrated with FDM scaffolds [20]. However, little attention is paid to these aspects in the design process of MEW scaffolds. In this study, we evaluate the effect of collector speed on scaffold properties, with a particular focus on crystallinity and resulting mechanical properties.

**FIGURE 1:**
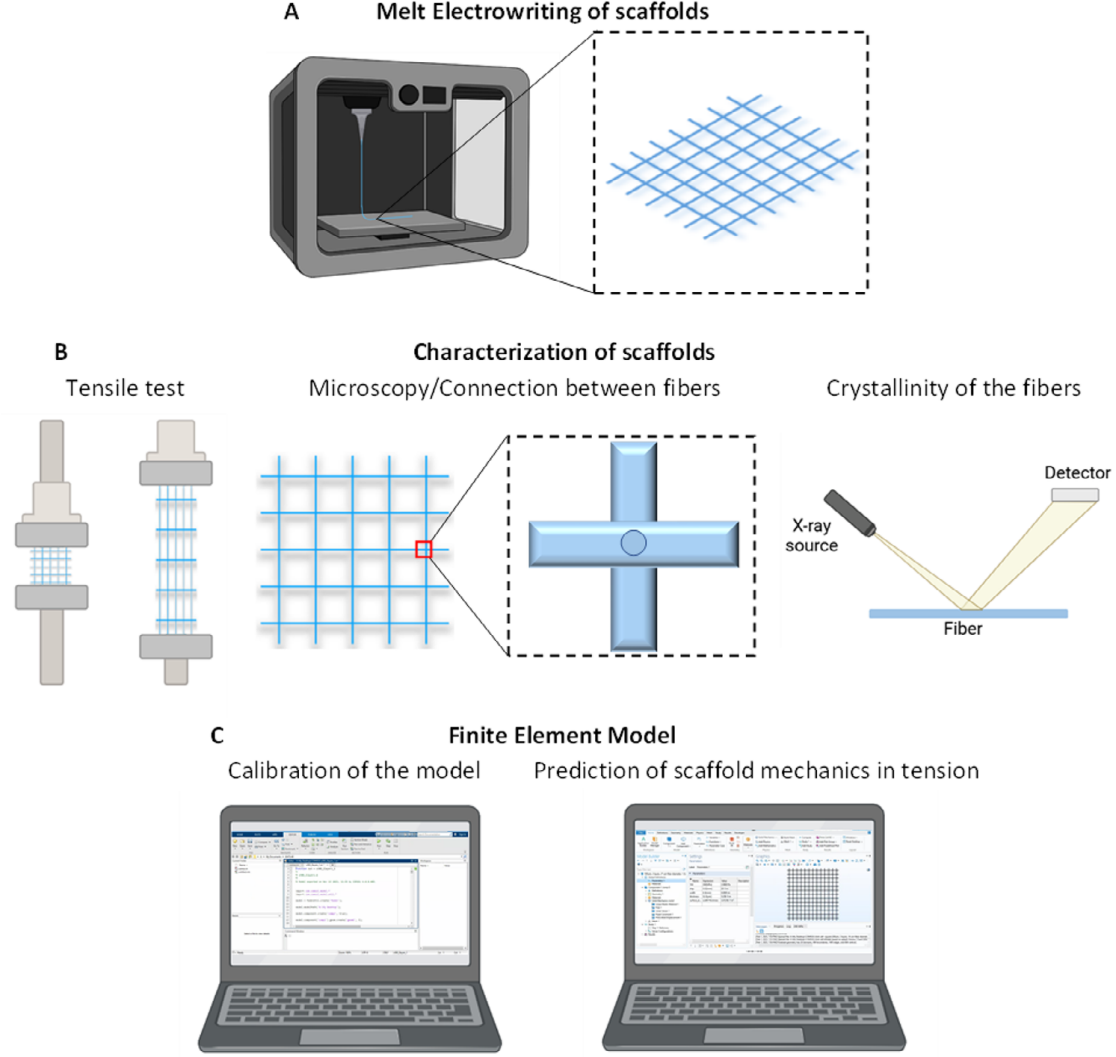
Schematic overview of the research workflow. (A) Melt Electrowriting of scaffolds. (B) Characterization of printed scaffolds, including tensile testing, microscopic visualization to asses printing quality and connection between fibers, and wide‐angle X‐ray scattering to analyze the crystallinity of the printed fibers. (C) Development of the finite element analysis model by comparing experimental with in silico results, and simulating scaffold behavior in tension.

While the trial‐and‐error method remains the most common approach in the scaffold design process, tools that predict the mechanical properties of the printed construct are increasingly being implemented. Models based on finite element analysis (FEA) are a promising method to predict the mechanics of scaffolds before printing, allowing the simulation of complex geometries and material behaviors. Although there are already well‐developed FEA models for scaffolds produced using FDM [21, 22], for MEW scaffolds, so far only, a few models have been proposed [23, 24], and they do not include the effect of printing parameters (e.g., collector speed) on the mechanical properties of the printed material. Here, we develop and calibrate an FEA model tailored to assess scaffold mechanical performance under tensile loading conditions for many different designs, for example, square, rhombus, or radial (Figure 1). Tensile testing was chosen as the primary mode of mechanical characterization, given its widespread use for MEW scaffolds and relevance in tissue engineering approaches, for example, when culturing under uniaxial dynamic stimulation.

## 2 Methods

### 2.1 Fabrication of Scaffolds

The MEW printer (The MELT by Spraybase A‐1204‐0001‐01D) was used to fabricate all the scaffolds. Printed designs were generated using the SEL Program Generator (IAI Corporation, Japan): square, rhombus, triangular, and radial with different numbers of layers, fiber diameters, and lay‐down angles (angle between fibers in the consecutive layers), as abbreviated in Table 1. For the square and triangular designs, the number in the sample name represents the inter‐fiber distance. In the case of rhombus designs, the specified angle refers to the angle direction of the tensile test. For radial designs, the amplitude of the wavy fibers is indicated. “FD” in the sample description corresponds to the fiber diameter. The inter‐fiber distance in rhombus designs was 200 µm, while for radial designs was 500 µm. Medical‐grade polycaprolactone (PCL, PURASORB PC 12, Corbion, the Netherlands) was used for printing. Before the extrusion process, PCL was loaded to the printing cartridge and heated for 1 h to ensure homogenous melting of the material. To stabilize the polymer jet, the sacrificial zone consisting of parallel lines was printed for 3–5 min, prior to the scaffold production. Collector speed, pressure, and voltage applied were adjusted to the particular design, as presented in Table 1. Each scaffold was printed with eight layers unless stated otherwise and an overall size of 3 × 3 cm.

**TABLE 1:** Printing parameters of the scaffolds.

		Printing parameters					
Abbreviation	Number of layers	Voltage (kV)	Pressure (bar)	Collector speed (mm/min)	Temperature (°C)	Working distance (mm)	Nozzle diameter (mm)
Square 300 µm (FD 8 µm)	8	4.90	0.030	600	100	4	0.3
Square 300 µm (FD 8 µm)	8	4.50	0.045	1500	100	4	0.3
Square 300 µm (FD 8 µm)	8	6.00	0.200	4800	100	4	0.3
Square 200–500 µm (FD 16 µm)	8	6.00	0.150	1500	100	4	0.3
Triangular 200 µm (FD 16 µm)	6	6.00	0.150	1500	100	4	0.3
Rhombus 42–120° (FD 16 µm)	8	6.00	0.150	1500	100	4	0.3
Radial 110–300 µm (FD 16 µm)	8	4.8–6^a^	0.070	540	100	4	0.3

^a^ Due to the changes in humidity, the applied voltage needed to be adjusted when printing radial design.

### 2.2 Scaffold Characterization

#### 2.2.1 Scaffold Visualization

The printed scaffolds were imaged with a light microscope (Nikon SMZ‐2T, Japan) equipped with a transmitted light table and a ToupCam camera (UCMOS5100KPA 5 MP CMOS, ToupTek, China). The fiber diameter was measured using ToupTek ToupView 3.7 software. At least 10 fibers per scaffold were measured, and three scaffolds of each type were analyzed. The average fiber diameter was calculated.

For SEM investigation, the scaffolds were fixed on a silicon wafer using a metal ring. Next, they were coated with gold using a JEOL JFC‐1300 auto fine coater at 30 mA for 60 s. SEM visualization was conducted using an FEI Quanta 400 FEG in high vacuum mode at accelerating voltages of 3 and 10 kV. Both secondary electron (SE) and backscattered electron (BSE) images were obtained using the Everhart‐Thornley (ETD) and Solid‐State Detector (SSD).

#### 2.2.2 Mechanical Characterization of Scaffolds

Uniaxial tensile tests were performed on all printed scaffolds to evaluate their mechanical properties using a universal testing machine (Instron 34SC‐1KN, Instron, USA) with a 5 N load cell. To ensure precise control over fiber orientation during testing, the scaffolds were first cut under a microscope using a scalpel into rectangular shapes (approximately 10 mm in width and 10 mm in length). After cutting, the samples were attached to a paper frame using double‐sided tape under the microscope, ensuring the fibers were aligned in the desired direction. This step prevented any misalignment or shifting of the samples during placement into the pneumatic clamps (with a pressure of 1 bar to avoid slippage), ensuring the fiber orientation remained consistent and as intended. Right before the tensile test, the sides of the paper frame were carefully cut using scissors. The tensile test was performed at a constant speed of 5 mm/min at room temperature until breakage appeared. The strain‐stress curve of each scaffold was analyzed. Young's modulus (*E*) was calculated based on the stress‐strain curve at the initial linear part (strain varying based on the design, up to a maximum of 150 %) of the curve using Equation (1):

(1) $$E=\frac{\sigma_{2}-\sigma_{1}}{\varepsilon_{2}-\varepsilon_{1}}\;$$

where *σ*_1_ and 𝜎_2_ are the stress values at two points on the linear portion of the stress‐strain curve, and 𝜀_1_ and 𝜀_2_ are the corresponding strain values at those two points. The force at break was chosen based on the maximum recorded force. Three samples of each design consisting of eight layers, unless stated otherwise, were tested.

#### 2.2.3 Wide Angle X‐Ray Scattering

The measurements were performed at the MINA diffractometer of the University of Groningen, equipped with a Cu rotating anode emitting X‐ray wavelength at 1.5413 Å (corresponding to 8 keV), and a Bruker Vantec 500 2D detector with a pixel size of 136 × 136 µm and a beam size of 250 × 250 µm (HxV). Sample‐to‐detector distance of 80 mm was used for experiments in transmission mode. Depending on the scattering power of each sample, the acquisition times were varied from 200 to 240 min in order to minimize signal‐to‐noise ratios and collect high‐quality data.

The obtained patterns were converted into 1D intensity profiles using the freeware Fit2D (ESRF, Grenoble, France), and the pixel scale was converted into the q scale (with q=4πsinθ/λ) using the known peak positions from a standard silver behenate sample. The background of the empty chamber containing the sample was subtracted after proper scaling for the sample absorption.

The crystallinity degree ($\chi_{c}$) was calculated as:

(2) $$\chi_{c}=\frac{\sum A_{c}}{\sum A_{c}\sum A_{a}}\;$$

where *A_c_* and *A_a_* are the fitted areas of the crystalline and amorphous phases, respectively. A linear combination of Voigt functions, implemented in a homemade MATLAB code, was used to reproduce the different WAXS signals arising from each crystalline peak and the amorphous halo.

#### 2.2.4 Connection Between Fibers

Eight‐layered scaffolds with rectangular (500 µm × 1000 µm) and rhombus (lay‐down angle 42°, inter‐fiber distance 700 µm) pore shapes have been printed to investigate the connection between fibers at the nods. To separate fibers at the nodes, the scaffolds were placed under the stereo microscope and individual fibers were detached using tweezers. Next, the bond footprint of the connection between two fibers was imaged using SEM. The diameter of the footprint of the detached fiber was measured using ImageJ. The diameter was measured for 20 footprints for each scaffold. A similar procedure was done for scaffolds printed with the same overall architecture (pore size 300 µm, fiber diameter 8 µm, two layers) but printed at different collector speeds, namely 600, 1500, and 4800 mm/min, to check the influence of different collector speeds on the cooling rate of the fibers.

### 2.3 Finite Element Analysis

The numerical simulations were carried out using the Structural Mechanics Module of COMSOL Multiphysics. In the simulations, we assumed a perfect, defect‐free geometry and used solid 3D finite elements to reproduce the finite‐element models of scaffolds. Cylinder structures were used to recreate the fibers. The unit cell in the model is regarded as a single pore geometry. The number of unit cells varied between 135 and 150 in every model, and the models were symmetric to minimize the influence of boundaries on the simulation results. The connection area between fibers is set at 0.5 µm for 4800 mm/min, and 1.5 µm for 600 or 1500 mm/min, based on the SEM imaging. The bulk material (PCL) is assumed to be isotropic with a mass density of 1145 kg/m^3^, Poisson's ratio of 0.3 (Figure S1), and Young's modulus of 168 or 265 MPa for scaffolds printed at 4800 mm/min, and 600 or 1500 mm/min, respectively. Linear elastic material behavior was implied, ignoring viscoelastic effects. To reproduce tensile test conditions, one end of a scaffold model was fixed, and a prescribed displacement was applied to the opposite end. Free tetrahedral mesh was applied with a fine mesh size, and the mesh convergence analysis was performed. Geometric nonlinearity was considered in the simulations. Young's modulus was calculated based on the stress values from simulations at two points from the linear range of the stress‐strain curve (*σ*_s1_ and 𝜎_s2_), and the corresponding strain values at those two points (𝜀_s1_ and 𝜀_s2_) as follows:

(3) $$E=\frac{\sigma_{s2}-\sigma_{s1}}{\varepsilon_{s2}-\varepsilon_{s1}}\;$$

The numerical results were calibrated by comparison to the experimental data obtained from the tensile tests of corresponding scaffold geometries.

### 2.4 Calibration of Bulk Young's Modulus

In this work, the used linear elastic material model requires two material parameters: Young's modulus and Poisson's ratio. Poisson's ratio is determined according to literature. Young's moduli corresponding to different collector speeds of 600, 1500, or 4800 mm/min are determined utilizing optimization techniques, which avoid trial‐and‐error efforts and enhance accuracy. The first step is to simulate the experimental loading conditions with an initial guess of Young's modulus within the 150–300 MPa range. Then, the model predictions are compared with the average experimental results, and a scalar error value of the predictions is calculated. Finally, an optimization algorithm is used to determine a new choice of Young's modulus, and the procedure is repeated until the error value is smaller than the expected value of 0.02 [49]. The parametric optimization is implemented in Isight combined with numerical simulations in COMSOL, where MATLAB code acts as the link. Specifically, the MATLAB code uses the new choice of Isight as input and transfers it to COMSOL to run the numerical simulations, thereafter the calculated results are forwarded to Isight again using the MATLAB code [57]. To validate the optimized bulk Young's modulus values obtained from Isight, they are applied to different numerical scaffold models. The numerical predictions for Young's modulus are compared with the corresponding experimental results and the minimal difference is used to choose the best fit.

### 2.5 Statistical Analysis

All samples were tested in triplicates. Statistical analysis was performed using GraphPad Prism 8 (GraphPad Software Inc., USA). The mean value ± standard deviation was reported for all data. Comparison between data was analyzed by ANOVA with Tukey's post hoc test. Results were considered significantly different at *p ≤ 0.05, **p ≤ 0.01, ***p ≤ 0.001, and ****p ≤ 0.0001.

## 3 Results and Discussion

### 3.1 Crystallinity and Mechanical Properties

**FIGURE 2:**
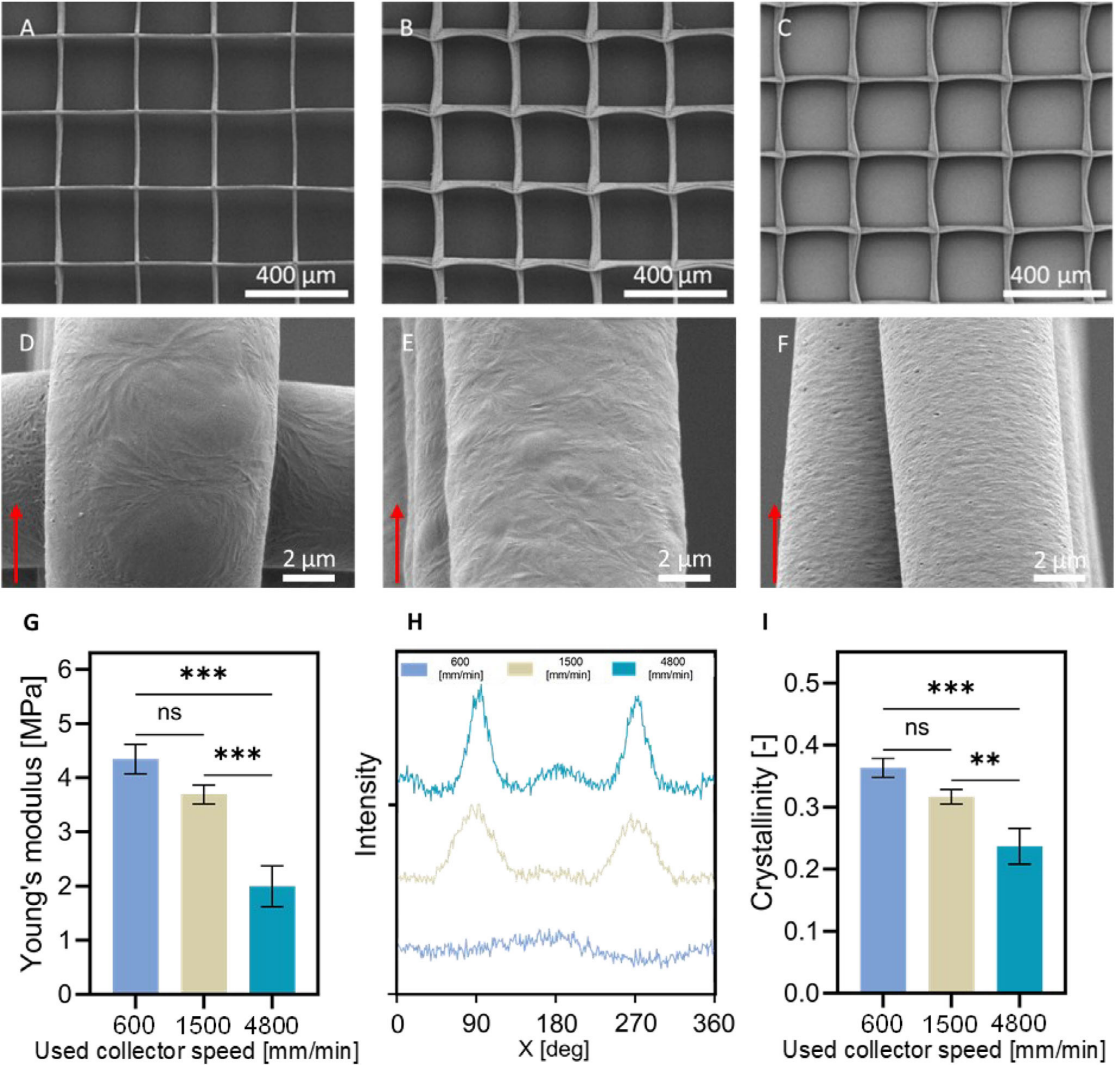
Effect of the used collector speed on properties of the printed structures. SEM images of scaffolds printed with a pore size of 300 µm and 9 µm fiber diameter with different collector speeds (A, D) 600 mm/min, (B, E) 1500 mm/min, and (C, F) 4800 mm/min. Red arrows indicate the direction of fiber laydown. (G) Young's modulus of MEW scaffolds printed at different speeds but with the same overall architecture. (H) Azimuthal WAXS scans revealing increased structural anisotropy and (I) crystallinity of the fibers.

MEW was used to print scaffolds with the same architecture (300 µm square pore shape with 9 µm fiber diameter) using different collector speeds, namely 600, 1500, and 4800 mm/min (Figure 2). To maintain a constant fiber diameter while increasing the collector speed, the applied pressure was adjusted to 0.03, 0.065, and 0.2 bar, respectively. Macrostructural examination via Scanning Electron Microscopy (SEM) revealed consistent pore shape and precision across all scaffolds, irrespective of collector speed (Figure 2A–C). However, upon closer look at the fibers of printed scaffolds, alterations in fiber morphology could be seen. Namely, a clear transition from spherulites to parallel lamellae on the surface of the fiber was visible with an increase in collector speed (Figure 2D–F). Tensile testing of scaffolds with identical overall architecture revealed a decline in mechanical properties with increased collector speed (Figure 2G). Specifically, Young's modulus exhibited a reduction of over 40% as the speed increased from 600 to 4800 mm/min for a 9 µm fiber size. Importantly, no statistically significant difference was observed between scaffolds printed at speeds of 600 and 1500 mm/min. Previously, only in one study conducted by Blum et al., a similar relationship between collector speed and its impact on scaffold structures was observed, although limited to collector speeds lower than those examined in this research (up to 1763 mm/min) [19]. The authors reported a decrease in Young's modulus, assessed via atomic force microscopy, with increasing collector speed (from 484 to 1763 mm/min); however, no statistical significance was observed. Notably, our study extends this investigation to higher collector speeds, revealing a consequential impact on scaffold mechanical properties.

To shed light on the observed variation in Young's modulus of printed scaffolds for the different collector speeds, wide‐angle X‐ray scattering (WAXS) analysis of printed fibers was conducted. Using WAXS, we aimed to determine the effect of collector speed on fiber crystallinity and chain axis orientation, hereafter referred to as molecular orientation. The WAXS patterns of the fibers revealed an increased structural anisotropy when the collector speed was increased. As the collector speed increased, the intensity of the crystalline rings shifted from being evenly distributed to becoming more focused into arches, as a result of the increased crystallite orientation (Figure S2). Figure 2H reports the azimuthal intensity curves of the (110) diffraction peak for the PCL unit cell, where the azimuthal angles (*χ*) of 0° and 180° are defined as the fiber axis direction. As the collector speed increases, two maxima at 90° and 270° appear and become sharper and more intense by revealing an increase in the orientation of the chain axis along the fiber axis (axial orientation). The change in molecule orientation could also be observed in SEM images as the fiber surface changed from spherulites to parallel lamellae (Figure 2D–F). This transformation was the effect of increasing stretching of the melted polymer jet which resulted in increasing orientation of the macromolecules [25, 26].

The crystallinity of the fibers, determined by WAXS, decreased with increasing collector speed from 36 ± 2% to 24 ± 3% for 600 and 4800 mm/min, respectively. The increased stretching of the fibers due to higher collector speed further led to the increasing orientation of molecules (Figure 2I). This trend contradicts findings from other studies that reported enhanced crystallization with increased orientation of the molecules [27, 28]. However, previous studies primarily utilized FDM, which prints typically much thicker fibers, where changes in cooling rate have a much higher influence on molecules orientation than stretching (due to a higher mass of printed fibers and lack of electrical force). In the case of MEW fibers, as collector speed increases, the polymer jet remains suspended in the air longer before deposition, leading to a higher cooling rate and consequently decreasing the crystallinity of the fibers, yet, with an increase in stretching and, therefore, molecular orientation. The different cooling rates of fibers printed with different collector speeds were further confirmed by the connection area between fibers at the nodes. As the collector speed increased, the connection area between fibers decreased. This indicates that fibers printed at higher collector speeds were cooled faster, which contributed to the decrease in crystallinity. Thomas et al. also reported that as the collector rotation speed was increased from 0 to 6000 rpm during the electrospinning process, there was a corresponding decrease in the crystallinity of the PCL fibers. They attributed this phenomenon to the rapid stretching of the fibers during electrospinning which in turn prevented the polymer chains from having sufficient time to fully crystallize [29]. Interestingly, the decrease in crystallinity with increasing strain was also observed in a study done by Kamal et al., who performed WAX measurements during the tensile test of hot‐pressed PCL samples [30].

The WAXS results showed the clear dependence of fiber crystallinity on the collector speed, and therefore, the influence of collector speed on the mechanical properties of the scaffolds. Although much research showed the importance of different printing parameters on MEW scaffolds, this significant and strong effect of collector speed was not considered before in the experimental studies using MEW. We argue that this relation should not be overlooked. The controlled manipulation of crystallinity in MEW scaffolds has not only impact on tensile modulus, but also opens up new exciting possibilities, for example, in drug release systems. Higher crystallinity may provide sustained drug release, while lower crystallinity could enhance initial burst release, making it a versatile platform for tailoring drug delivery profiles to specific therapeutic needs [31]. Notably, the ability to influence crystallinity through collector speed offers a more direct and efficient means of achieving desired drug release profiles, compared to the various post‐treatment methods that have been employed including annealing, solvent exposure, or thermal treatments [32, 33, 34]. Crystallinity can also influence the degradation properties of the polymers. Less crystalline regions are more susceptible to hydrolytic degradation due to the water molecules penetration and breaking of chemical bonds more easily [35]. For instance, in the study on PLA substrates, those with lower crystallinity demonstrated decreased resistance to enzymatic degradation [36]. The significant impact of crystallinity on cellular behavior and differentiation was also reported previously. Cell adhesion of fibroblasts was shown to be higher on the more crystalline zones of polylactic acid (PLA) films [37]. In another study, PLA scaffolds were fabricated using the FDM technique to achieve varying levels of crystallinity: one set with low crystallinity (∼8%) and another with higher crystallinity (∼18%). The findings revealed that human mesenchymal stromal cells seeded on scaffolds with higher crystallinity exhibited a notable increase in proliferation rate and expressed a higher level of osteogenic differentiation marker by day 21, in contrast to those on scaffolds with lower crystallinity content [38]. Jiafeng et al. reported similar results, observing higher osteoblast activity on PLA scaffolds with higher crystallinity context (31.2%) [39]. In another study, it was proven that fibroblasts seeded on PCL substrates with higher crystallinity expressed higher levels of collagen and Alpha‐smooth muscle actin, which are myofibroblast markers [40]. These findings underscore the important interplay between polymer crystallinity, degradation behavior, and cellular responses, highlighting the importance of tailoring material properties for optimized tissue engineering outcomes.

### 3.2 The Connection Area Between Fibers

**FIGURE 3:**
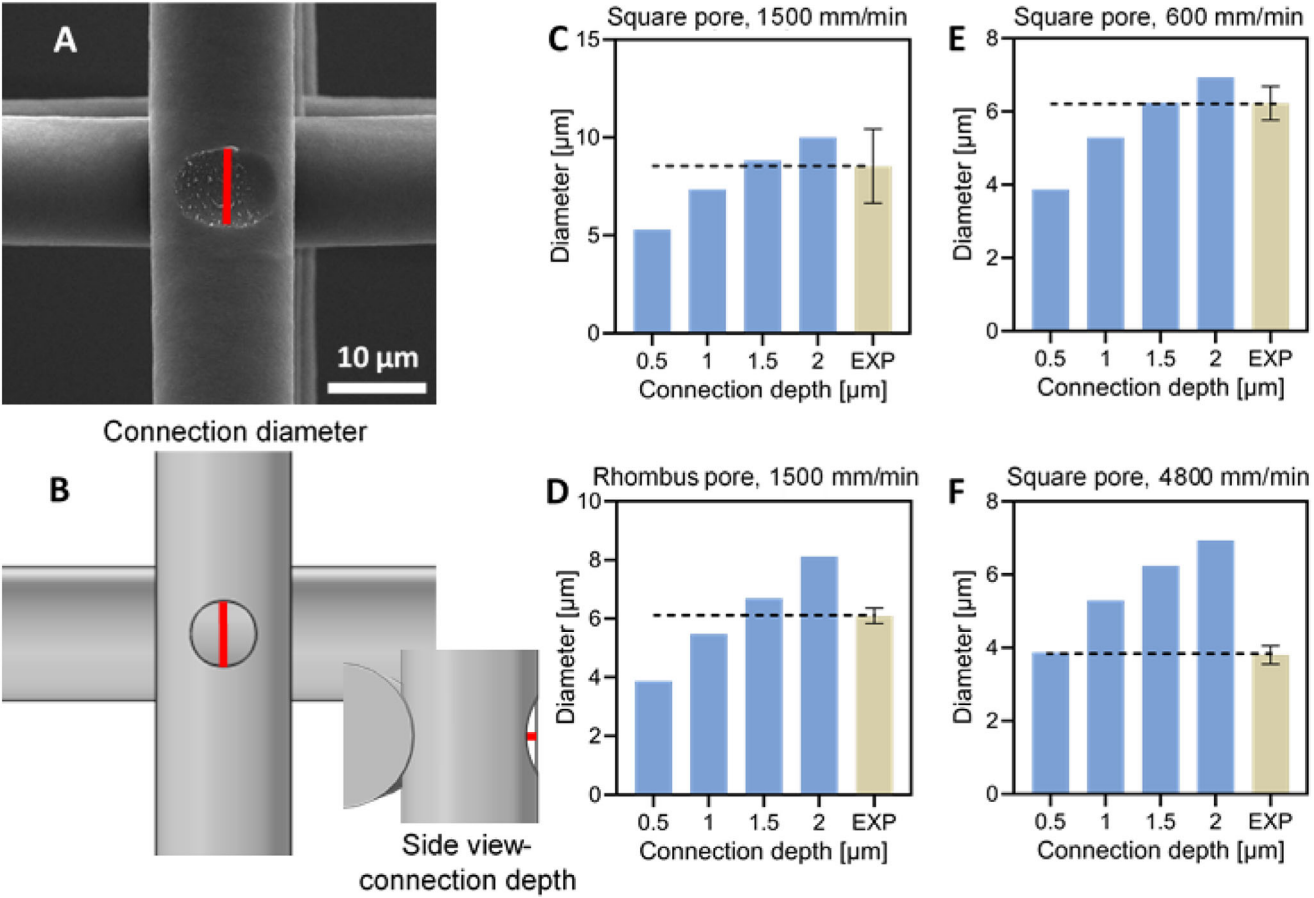
The size of the connection area between fibers. (A) SEM image of the footprint of the upper fiber in the fiber below printed at 100°C. (B) The footprint in the numerical model: top and side view of the footprint. The red lines on the images represent the measured diameter (top view) and the connection depth (side view) of the connections between fibers. (C, D) The comparison between diameters measured for different connection depths set up in the prediction model (blue columns) with a measured diameter of connection size between fibers for printed scaffolds (yellow column) for square design, and for rhombus design printed at 1500 mm/min, respectively. (E, F) The comparison between diameters measured for different connection depths set up in the prediction model (blue columns) with a measured diameter of connection size between fibers for square mesh scaffolds (yellow column) printed with the same fiber diameter (8 µm) but at different collector speeds of 600 and 4800 mm/min, respectively.

The connection area between fibers refers to the degree to which the fibers in consecutive layers are merged at the nodes after printing, as a hot melted polymer is deposited on a previously printed layer. It is another parameter that can be influenced by collector speed and cooling rate and holds significant importance for the final scaffold's properties. It affects the structural stability and, thus, mechanical properties of the overall structure. To estimate the connection area, we employed SEM. We examined the influence of various collector speeds (600, 1500, and 4800 mm/min), and two different pore shapes (square and rhombus) (Figure 3). The area was measured by detaching the upper fiber, and then determining the diameter of the resulting footprint on the lower fiber (Figure 3A). For scaffolds with different pore shapes but identical fiber diameters, the connection depth was found to be the same (Figure 3C,D). This indicates that the shape of the pores does not play a major role in determining the connection size between fibers. As the collector speed increased from 600 to 4800 mm/min, the diameter of the connection area decreased from 6.2 ± 0.5 µm to 3.8 ± 0.3 µm, respectively (Figure 3E,F). This we attributed to the different cooling rates for fibers printed at different speeds [41, 42].

Lamb et al. also investigated the fusion of PCL fibers printed using MEW, examining the impact of collector speed on fiber bonding. They found that increasing the collector speed from 300 to 600 mm/min enhanced fiber bonding by reducing the cooling time between consecutively deposited fibers. However, in their study, the jet lag remained constant across different speeds. Therefore, the fibers printed at higher speeds were deposited more quickly on top of the already printed ones, providing less time for the extruded material to cool and thereby promoting better fusion between the layers. In contrast, in our study, as the collector speed increased, the jet lag also increased, resulting in longer suspension times for the fibers in the air before deposition. This increased suspension time led to the opposite effect, namely increased cooling of the fibers, reducing the connection area between them. Despite these differences in cooling dynamics, both studies highlight the importance of the time between fiber extrusion and deposition in determining bonding strength, which ultimately affects the mechanical properties of the scaffolds. Lamb et al. also reported a 70% increase in layer bond yield strength when the collector speed increased from 300 to 600 mm/min, underscoring the crucial role of fiber bonding in the overall mechanical strength and structural integrity of the scaffold, alongside its geometric features [43]. Additionally, a higher degree of bonding between fibers increases fiber density, which in turn influences the material's mechanical properties.

### 3.3 FEA Model

After examining the intricate interplay between crystallinity, connection area between fibers, and mechanical properties, we developed an FE model tailored to characterize the mechanical behavior of MEW scaffolds. Our objective was to construct a predictive model capable of accurately estimating scaffold mechanical properties prior to the printing process. The focus of the FEA model was on the tensile loading of MEW scaffolds. This approach was chosen due to the enduring popularity of tensile testing as a method for assessing the mechanical properties of MEW scaffolds, especially given its relevance in scenarios where tensile force is applied to the MEW scaffolds during cell culture stimulation [44]. To develop the FEA model, we made several assumptions regarding the scaffold's geometry and material properties. We assumed that the printed scaffold has a perfect, defect‐free geometry, and therefore, we used solid cylinders to reproduce extruded filaments. The bulk material was assumed to be isotropic, homogeneous, and linear elastic.

In the first step, we used the FE model to compare the averaged measured diameter of the footprint obtained experimentally with data from numerical simulations. The numerical model considered fibers of 8 µm diameter. To estimate the connection depth for the printed scaffolds, the diameter of the footprint in the numerical model at a connection depth ranging from 0.5 to 2 µm was measured. The obtained values were compared with the corresponding diameters measured experimentally. The numerically estimated diameter of the footprint at a collector speed of 600 and 1500 mm/min was equal to the one set at 1.5 µm in the numerical model (Figure 3C,E). For a collector speed of 4800 mm/min, it was equal to 0.5 µm (Figure 3F). Therefore, a connection size of 0.5 µm was used in a model of the scaffold obtained with a collector speed of 4800 mm/min, and 1.5 µm for a collector speed of 600 and 1500 mm/min. It is worth noting that fiber sagging during printing results in additional connections between fibers (for more details, see Figure S3) [45]. However, to simplify the model, only the intentional connections at the crossings were considered. Sagging of the fibers was excluded from the FEA model as it did not affect the fiber orientation in the tensile direction, implying that it would not significantly influence mechanical properties like tensile strength or stiffness, which are determined by fiber alignment and material density along the load axis. It is important to mention that the connection depth was estimated using the FEA model, which will be discussed in subsequent sections in detail.

#### 3.3.1 Configuration of the Fibers

**FIGURE 4:**
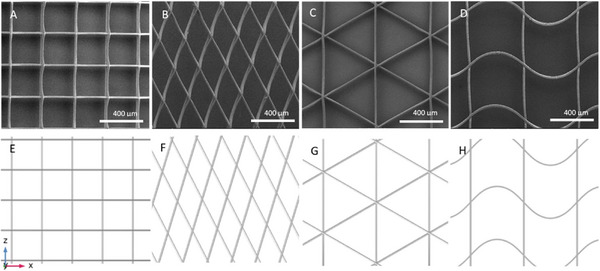
Comparison between SEM images of PCL scaffolds and built geometric models. (A‐D) SEM of the scaffolds with square, rhombus, triangular, and radial pore shapes. (E‐H). The respective numerical models were created in COMSOL Multiphysics of different scaffolds.

We modeled the scaffolds of four different architectures (pore shapes): square, rhombus, triangular, and radial (Figure 4). These shapes were selected because they have distinct responses to tensile load. They are also used in various tissue engineering applications (e.g., bone, skin, or muscle regeneration [46, 47, 48]), where the mechanical properties of the scaffold play a crucial role in tissue restoration. We varied fiber diameters and pore sizes.

#### 3.3.2 Young's Modulus of Bulk PCL for the FEA Model

The most important material property in the FE model is bulk Young's modulus. Young's modulus of PCL varies for different types of PCL (e.g., different molecular weight or medical vs. technical grade); and it is crucial to choose it correctly. Given the high variability of (bulk) material Young's modulus and its substantial influence on the mechanical behavior of a scaffold, the ideal approach would be to determine it experimentally. While tensile testing is a reliable method for estimating its value, conducting a tensile test on individual fibers of diameter of 8–30 µm posed several challenges. The inherent complexities include securely gripping the fibers without slippage, managing the delicate nature of the fibers (easy to destroy), and the potential for inaccuracies in measurements (due to the precision of the measuring tools, human error, or variability in the fibers themselves) [49]. Consequently, to ascertain the bulk Young's modulus for PCL, a fitting procedure of Young's modulus using experimental data was implemented [50].

**FIGURE 5:**
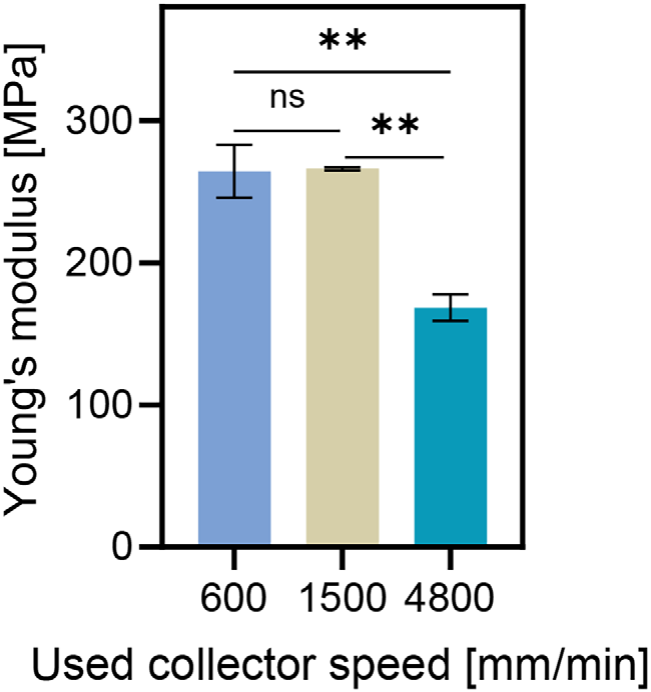
The relationship between the collector speed and theoretical Young's modulus of bulk PCL.

Employing the above fitting, we used numerical simulations to investigate how the bulk modulus of PCL changes for scaffolds printed at different collector speeds. The fitting outcomes revealed an increase in collector speed from 1500 to 4800 mm/min, corresponding to a reduced Young's modulus of bulk PCL from ca. 266 MPa to approximately 168 MPa. Interestingly, there was no significant difference in the bulk PCL Young's modulus when the collector speed increased from 600 to 1500 mm/min (Figure 5). This suggests that the mechanical properties of the printed PCL are influenced by collector speed, with higher speed yielding less rigid material. Moreover, this can indicate that there may be a threshold collector speed below which Young's modulus remains unaffected.

#### 3.3.3 Number of Layers

**FIGURE 6:**
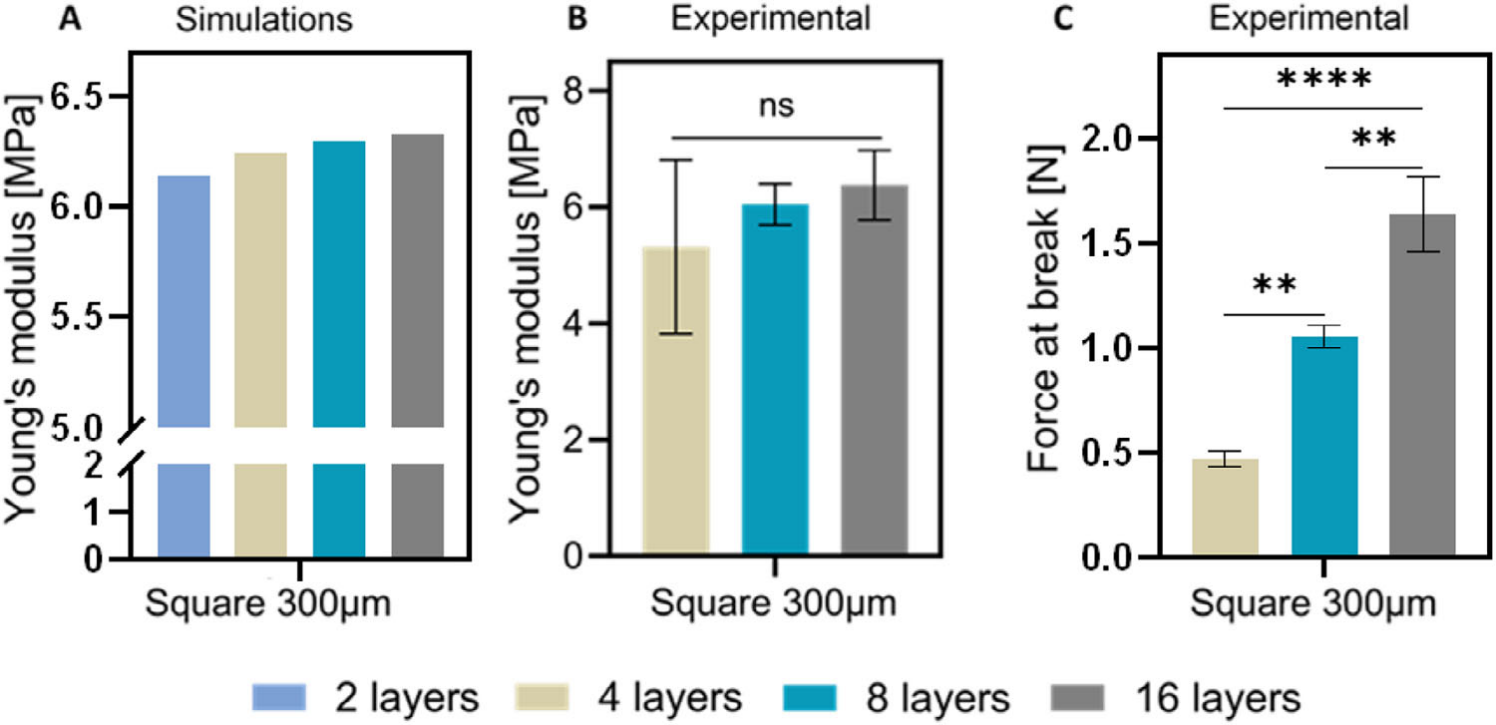
Mechanical properties of scaffolds with varying numbers of layers with a pore size of 300 µm and fiber diameter of 16 µm. Young's modulus of (A) numerical models and (B) printed scaffolds. (C) Force at break for scaffolds printed with varying numbers of layers.

Typical MEW scaffolds are composed of multiple polymer fibrous layers stuck on top of each other [51]. The computational simulations demonstrated an increasing stiffness by ca. 3% when the number of layers increases from 2 to 16, as reflected in the calculated Young's modulus (Figure 6A). This 3% increase in Young's modulus is negligible and we assumed that simulated Young's modulus is not dependent on the number of layers.

To confirm our conclusion on the independence of Young's modulus on the number of layers, scaffolds with the same pore sizes and shapes as in numerical models were printed and tested mechanically. For experimental settings, scaffolds with 4, 8, and 16 numbers of layers were chosen; scaffolds with only two layers were difficult to collect and handle during the tensile test. As can be seen in Figure 6B, the experimental Young's modulus of scaffolds remained constant regardless of the number of layers, so the same trend was observed from the numerical simulations. The independence of stiffness on the number of layers can be explained by the fact that the increase in the cross‐section area of the scaffold with the higher number of layers was balanced by a higher force needed for the scaffold deformation (Figure 6C). The small differences between the experimental Young's modulus for scaffolds with different numbers of layers can result from the sagging of the fibers: the more layers, the more sagging was observed. The data is consistent with the findings by Santschi et al., who reported no significant effect of the number of layers on Young's modulus of the MEW scaffold [52]. Moreover, few researchers reported decreasing printing accuracy with a higher number of layers which could also influence experimental results [10, 13].

Based on these results, we further analyzed scaffolds with only two layers (or three for triangular designs) to reduce computational load/power/time in the numerical simulations.

### 3.4 Calibration of FEA Model

**FIGURE 7:**
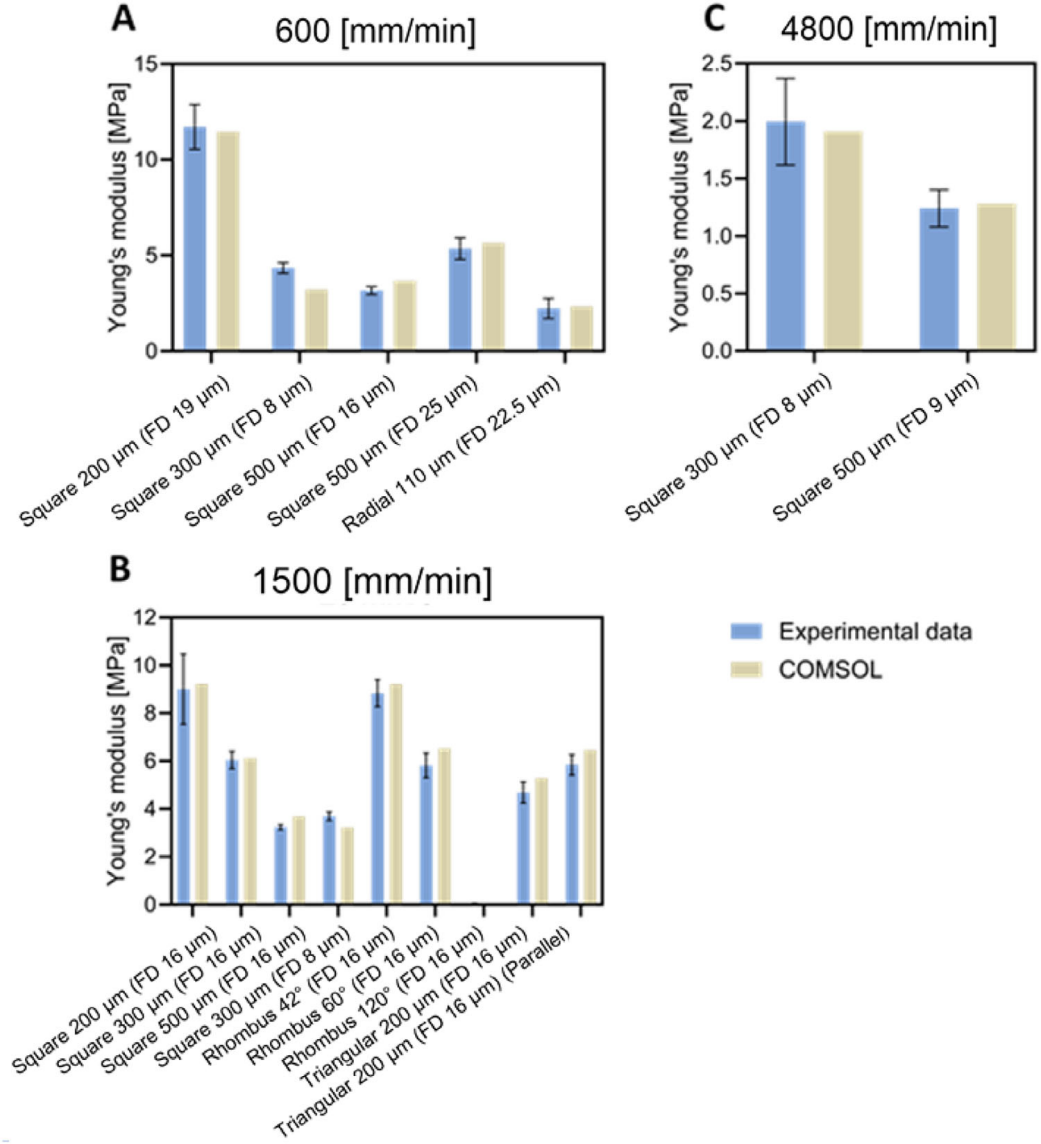
Calibration of the finite element analysis models.Comparison for scaffolds printed with (A) 600 mm/min, (B) 1500 mm/min, and (C) 4800 mm/min. All scaffolds consist of eight layers, except triangular scaffolds with six layers. Abbreviations are explained in Methods Section 2.1.

The numerical model was calibrated through the comparison with the tensile test experimental data. For this, the value of Young's modulus of bulk PCL for numerical simulations was chosen according to the collector speed (Figure 5). Next, we reproduced the tensile test conditions numerically for different designs of scaffolds, pore sizes, and fiber diameters (Figure S4) and found a strong correlation between the numerical and experimental data, independent of the design (Figure 7). This indicates that the calibrated model accurately captures the mechanical behavior of the MEW scaffolds subject to tension. The small discrepancies between the experimental and numerical results can be caused by the simplification assumptions for our numerical models, including idealized, defect‐free geometry, simplified fiber representation as ideal solid cylinders, and eliminated sagging of the fibers.

### 3.5 Insight into the Mechanics of MEW Scaffolds

Initial findings revealed an interesting influence of scaffold architecture on the mechanical properties of MEW scaffolds. Therefore, following the model calibration, the relationship between scaffold architecture and mechanical properties was investigated using both numerical simulations and experimental data from meshes with different pore sizes and shapes (square, rhombus, triangular, and radial). This section represents a step forward from the numerical model discussed in previous paragraphs.

#### 3.5.1 Square Pore Size

The simulations (Figure 7B), corroborated by experimental tensile tests (Figure S5A), indicated that decreasing the pore size of square scaffolds leads to an increase in Young's modulus. Smaller inter‐fiber distances required bigger forces to deform the sample, while the nominal cross‐section area of the whole sample did not change. A similar trend was observed before for MEW printed scaffolds [53, 54]. Furthermore, the change in the inter‐fiber distance in the direction perpendicular to the test direction did not influence Young's modulus (Figure S6).

#### 3.5.2 Rhombus Pore Size

In the rhombus designs, the decrease of the lay‐down angle in the direction of the tensile deformation resulted in a higher Young's modulus (Figure 7B). We observed an over 130‐fold increase in Young's modulus when the lay‐down angle decreased from 120° to 42°; and the increase from 0.1 to 9.2 MPa, for Rhombus 120° (FD = 16 µm) and Rhombus 42° (FD = 16 µm), respectively (collaborated by experimental data in Figure S5B). It can be explained by the fibers aligning in the initial phase of the tensile test, resulting in a higher strain before plastic deformation for scaffolds with a bigger lay‐down angle (stress‐strain curves shown in Figure S7A). These results are consistent with the previously published data indicating an increase in scaffold stiffness with increasing fiber alignment in the direction of the tensile test [55]. Further, the elastic region in rhombus designs increased from approximately 5% for Rhombus 42° (FD = 16 µm) to more than 150 % for Rhombus 120° (FD = 16 µm). Moreover, the scaffolds with a bigger lay‐down angle (> 60°) first exhibited the toe region before the stiffness started increasing in the elastic region. This pronounced toe region corresponds to a change in the fiber alignment, allowing better‐carrying tension load in scaffolds with bigger lay‐down angles [23]. Additionally, there was no statistical difference between Young's modulus of Square 200 µm (FD = 16 µm) and Rhombus 42° (FD = 16 µm); with the same inter‐fiber distance of 200 µm. However, the latter offered increased elastic properties which is observed as higher strain in the elastic region.

#### 3.5.3 Triangular Pore Size

The triangular design (Triangular 200 µm [FD = 16 µm]) tested in two different directions showed a difference in stiffness depending on the scaffold orientation; 5.3 and 6.5 MPa (schematic indicating the fiber alignment during the test for triangular design is shown in Figure S5C). This can be attributed to the stress distribution on the fibers during stretching. For triangular design with one layer perpendicular to the tensile test, most stress was localized on the fibers in the direction of the tensile load, while the horizontal fibers had a very small contribution to the stiffness. In the case of direction where one layer was in parallel to the tensile test direction, there were no fibers in the direction perpendicular to the applied tension, and this could cause a slight increase in the stiffness. When the triangular design was compared to the Rhombus 60° (FD = 16 µm), with the same angle between fibers, no statistical difference was observed. This suggests that an extra layer of fibers in a perpendicular direction to the force for a rhombus design does not influence Young's modulus due to different stress distribution on fibers oriented in different directions.

#### 3.5.4 Radial Pore Size

For the radial designs, a clear toe region was observed in the initial phase of the tensile test. It can be explained by the straightening of the wavy fibers. The value of the toe region is dependent on the amplitude of the wavy fibers: the bigger the amplitude, the bigger the toe region (Figure S7B), as observed previously [56]. The bigger amplitude of the radial design also resulted in a smaller Young's modulus (Figure S5D).

All modeled MEW scaffolds (except for those with square pore shapes) exhibited mechanical anisotropy. The most pronounced directional dependence of the mechanical properties was observed for rhombus and radial designs. For example, while testing Rhombus 60° (FD = 16 µm) in the direction of a smaller angle, the scaffold showed a high stiffness linear region (*E* = 6.5 MPa) in contrast to the direction of a bigger angle where the toe region could be observed (*E* = 0.1 MPa) (Figure S5B). Similar dependency was observed for the radial design; depending on the testing direction scaffolds exhibited an initial toe region (in the direction of wavy fibers) or a region with linearly increasing stress (in the direction of straight lines) (Figure S5).

Most importantly, it is essential to reiterate that, despite differences in scaffold architecture, the model is primarily based on the collector speed and the resulting cross‐sectional area (the connection between fibers). These two factors are critical in determining the mechanical properties, as they influence the material properties. The numerical simulations regarding the influence of the scaffold architecture on the mechanical properties were consistent with the data from the experimental tensile test. The analysis shows that the mechanical properties of MEW scaffolds can be tuned by a pre‐defined architecture, allowing fit‐to‐purpose designs.

## 4 Conclusions

This study takes a fresh view on MEW, demonstrating the significant influence of collector speed on fiber crystallinity and overall mechanical properties of MEW‐printed scaffolds of different designs. While traditionally regarded as a parameter primarily affecting fiber diameter and deposition patterns, our findings underscore its pivotal role in altering the material properties crucial for various tissue engineering applications. Consequently, our results advocate for a reevaluation of collector speed as a parameter, not only for geometric manipulation but also for tailoring material properties to specific application requirements. Altering the fiber crystallinity alone enabled changes in the scaffold's mechanical properties by approximately 2 MPa. This study also demonstrated the prediction model of the mechanical properties of MEW scaffolds in tension mode. SEM images of printed scaffolds show that the numerical models accurately represented the printed scaffolds in terms of their structure and geometry. It was observed that the connection depth between fibers should be set based on the applied collector speed; namely 1.5 µm for 600 and 1500 mm/min, and 0.5 µm for a speed of 4800 mm/min. Further, Young's modulus of bulk PCL, a parameter in the FEA model, showed dependency on the scaffold collector speed. By leveraging these findings, we were able to reveal a good correlation between experimental data and simulation results. We envision that the use of the proposed prediction model, will accelerate the production process and allow for the creation of MEW scaffolds with specific properties.

## Supporting information



Supporting information

## Data Availability

The data that support the findings of this study are available from the corresponding author upon reasonable request.
