# Supplementary material for: Designing Smartly: Understanding the Crystallinity of Melt Electrowritten Scaffolds
Source: Eng Life Sci. 2025 Apr 14;25(4):e70020. doi: 10.1002/elsc.70020 (PMC11997013; doi:10.1002/elsc.70020)
Supplement: Supplementary file 1 — Supporting information [file ELSC-25-e70020-s001.docx]

**Designing Smartly: Understanding the Crystallinity of Melt Electrowritten Scaffolds**

**Supplementary Information**

*Piotr Stanisław Zieliński^1^, Zhaohang Zhang^2^, Ilaria Squillante^3,4^, Guillermo Monreal Santiago^5^, Marcus Koch^6^, Giuseppe Portale^3,4^, Marleen Kamperman^1^, Anastasiia Krushynska^2^, Małgorzata Katarzyna Włodarczyk-Biegun^1,7*^*

^1^Polymer Science - Zernike Institute for Advanced Materials, University of Groningen, Nijenborgh 3, 9747 AG Groningen, The Netherlands

^2^Computational Mechanical and Materials Engineering - Engineering and Technology Institute Groningen, University of Groningen, Nijenborgh 4, 9747 AG Groningen, The Netherlands

^3^Physical Chemistry of Polymeric and Nanostructured Materials - Zernike Institute for Advanced Materials, University of Groningen, 9747 AG Groningen, The Netherlands

^4^Dutch Polymer Institute, P.O. Box 902, 5600 AX Eindhoven, The Netherlands

^5^Polymer Science - Zernike Institute for Advanced Materials, University of Groningen, Nijenborgh 3, 9747 AG Groningen, The Netherlands. Present address: Université de Strasbourg, CNRS, UMR7140, 4 Rue Blaise Pascal, 67081 Strasbourg, France

^6^INM- Leibniz Institute for New Materials, Campus D2 2, 66123 Saarbrücken, Germany

^7^Biofabrication and Bio-Instructive Materials, Biotechnology Center, The Silesian University of Technology, B. Krzywoustego 8, 44-100 Gliwice, Poland

*^*^*Corresponding author: Małgorzata K. Wlodarczyk-Biegun

E-mail: [m.k.wlodarczyk@rug.nl](mailto:m.k.wlodarczyk@rug.nl), [malgorzata.wlodarczyk-biegun@polsl.pl](mailto:malgorzata.wlodarczyk-biegun@polsl.pl)

### Poisson’s ratio of polycaprolactone

Poisson’s ratio of polymers is typically between 0.3 and 0.5. For polycaprolactone (PCL), the literature reports values between 0.3 and 0.442 [1, 2]. A numerical simulation was conducted to investigate the impact of different values of Poisson’s ratio on the tensile modulus of square and rhombus designs. The results showed that the influence of Poisson’s ratio was negligible, indicating that shearing during the deformation of scaffolds in the elastic region is minimal and can be disregarded (**Figure S1**). Consequently, a value of *ν*=0.3 for Poisson’s ratio, corresponding to a stiffer polymer [3], was selected for the final model.


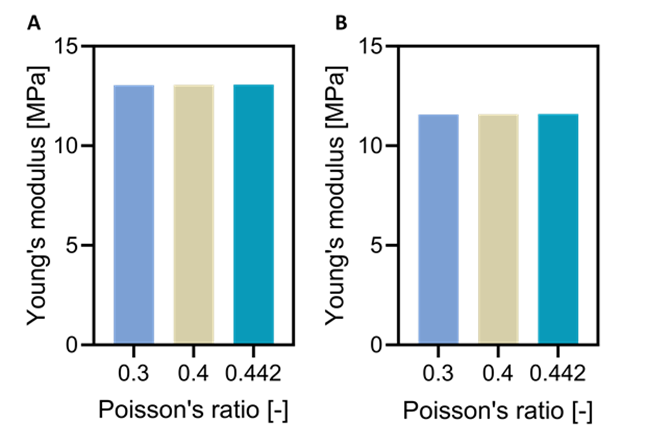


*Figure S1. Influence of different Poisson’s ratios on numerical simulation for models with (A) square and (B) rhombus pore size.*

*
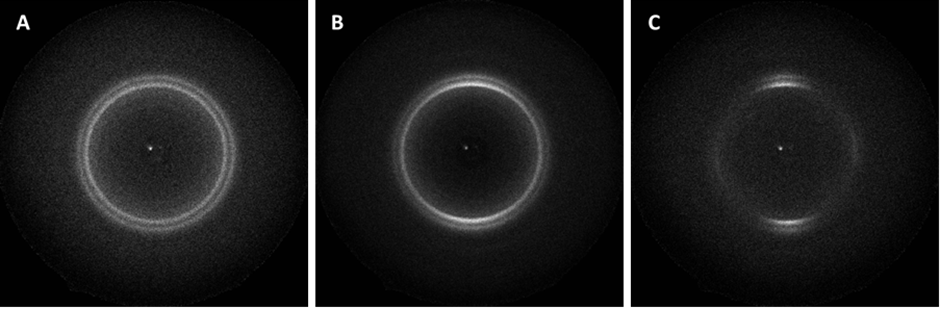
*

*Figure S2. 2D-WAXS patterns of MEW scaffolds printed at (A) 600 mm/min, (B) 1500 mm/min, and (C) 4800 mm/min.*


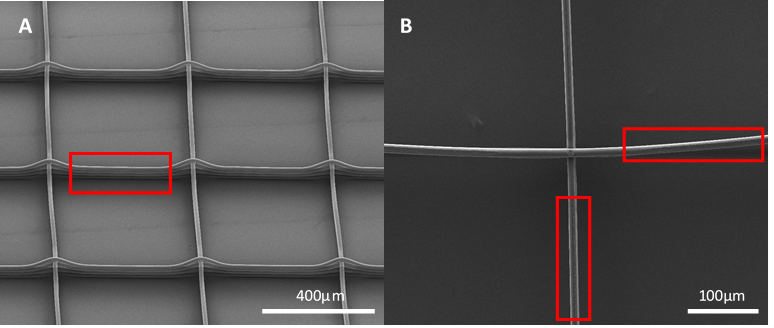


*Figure S3. SEM images of MEW scaffolds showing fiber sagging. (A) Overall view of the scaffold. (B) Close-up of the connection points of sagging fibers after fiber detachment. The connection of sagging fibers has been marked with red rectangles.*


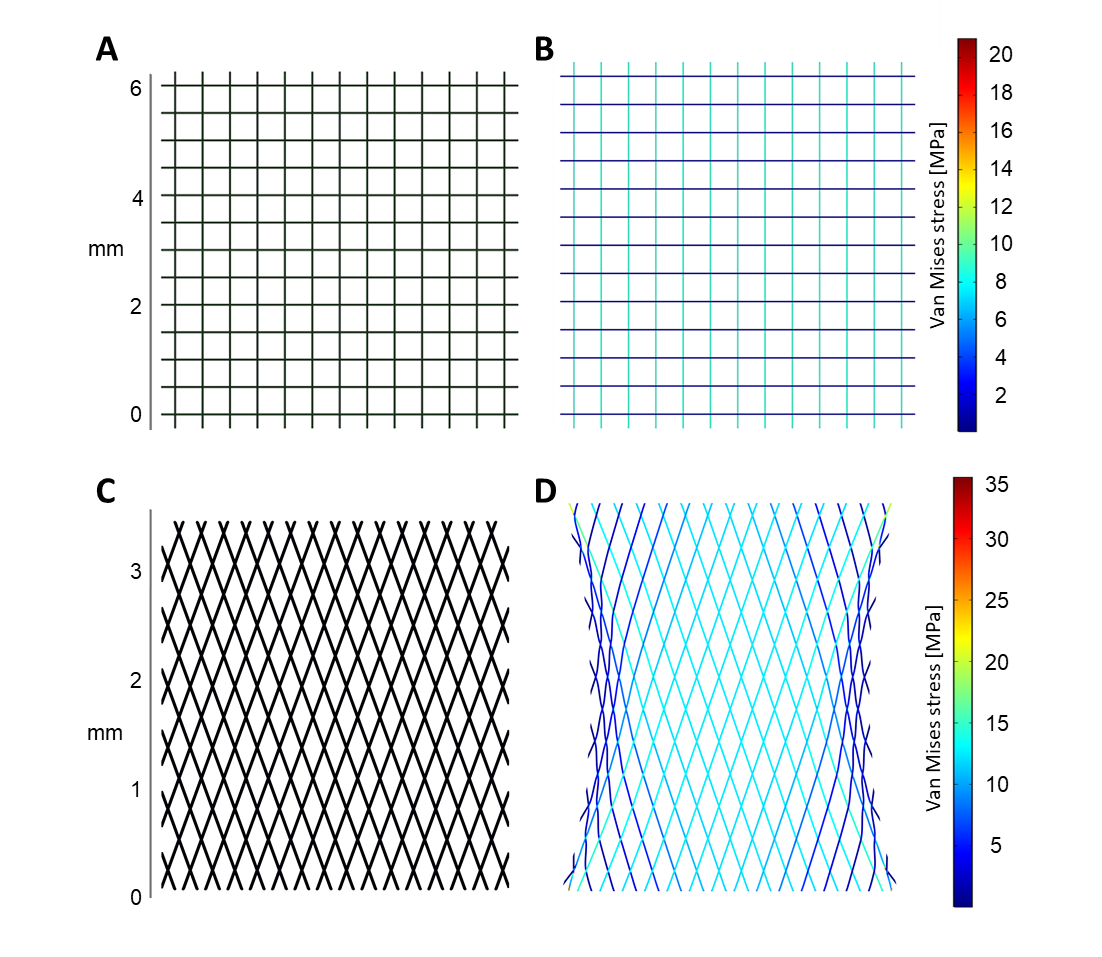


*Figure S4. Finite Element Analysis simulation results: (A) Square 500 µm (FD=25 µm) model before applying tension, (B) stress distribution at 3% tension, (C) Rhombus 42° (FD=16 µm) model before applying tension, and (D) stress distribution at 5% tension Both models have 2 layers. Abbreviations are defined in Methods Section 2.1.*


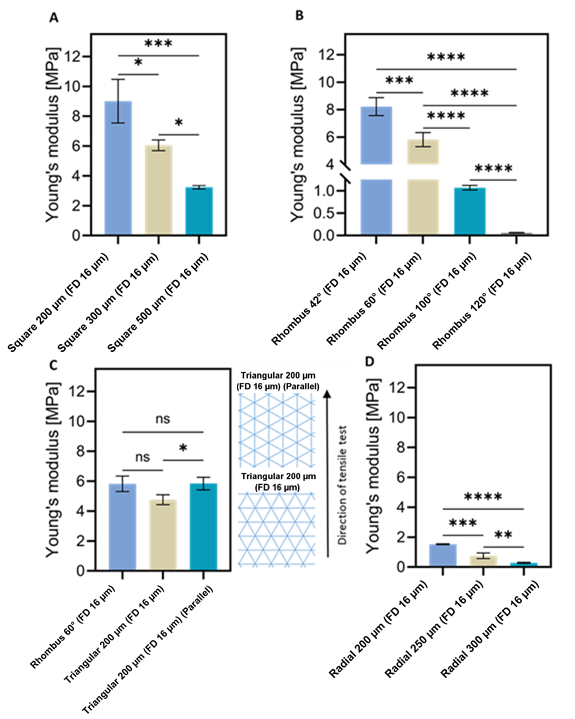


*Figure S5. Young's modulus of the scaffolds printed with MEW tested using tensile testing. (A) Scaffolds with square pores with different inter-fiber distances. (B) Scaffolds with rhombus pores with different lay-down angles in the direction of the tensile test. (C) Scaffolds with triangular pores tested in different directions with a schematic indicating the fiber alignment during the test. (D) Scaffolds with radial pores with different amplitudes of the fibers. Each scaffold consist of 8 layers, except triangular with 6 layers. Abbreviations are defined in Methods Section 2.1.*


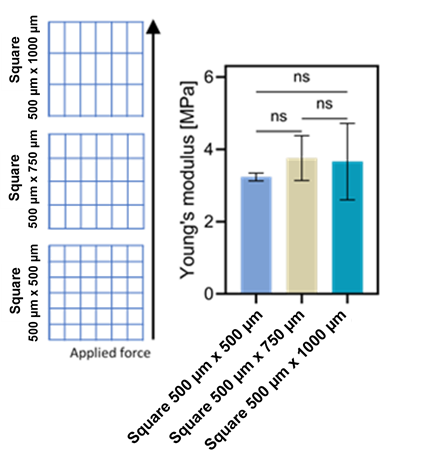


*Figure S6. Young’s Modulus of scaffolds printed with the same inter-fiber distance in the direction of the test (500 µm) and different inter-fiber distances in the direction perpendicular to the test (500, 750, 1000 µm).*


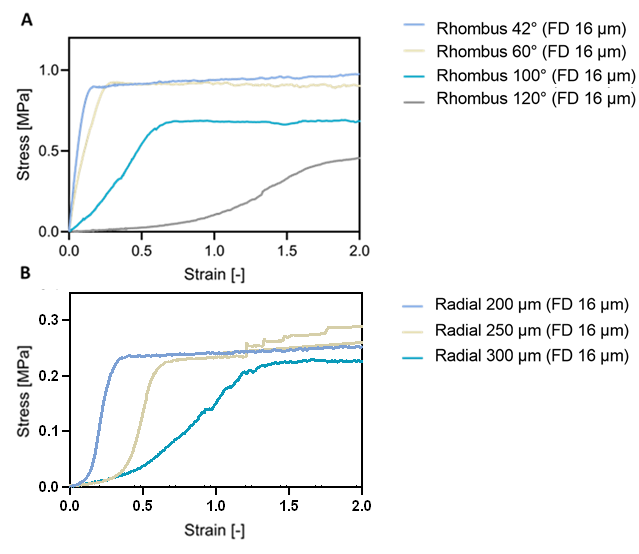


*Figure S7. Stress-strain curves for (A) rhombus designs with different lay-down angles and (B) radial designs with different amplitudes of the fibers. Each sample has 8 layers. Abbreviations are defined in Methods Section 2.1.*

### References

1. Eshraghi, S. and S. Das, *Mechanical and microstructural properties of polycaprolactone scaffolds with one-dimensional, two-dimensional, and three-dimensional orthogonally oriented porous architectures produced by selective laser sintering.* Acta Biomaterialia, 2010. **6**(7): p. 2467-2476.

2. Lu, L., et al., *Mechanical Study of Polycaprolactone-hydroxyapatite Porous Scaffolds Created by Porogen-based Solid Freeform Fabrication Method.* Journal of Applied Biomaterials & Functional Materials, 2014. **12**(3): p. 145-154.

3. Podichetty, J.T. and S.V. Madihally, *Modeling of porous scaffold deformation induced by medium perfusion.* Journal of Biomedical Materials Research Part B: Applied Biomaterials, 2014. **102**(4): p. 737-748.
